# Prevalence and Correlation of Anxiety, Insomnia and Somatic Symptoms in a Chinese Population During the COVID-19 Epidemic

**DOI:** 10.3389/fpsyt.2020.568329

**Published:** 2020-08-28

**Authors:** Yuanyuan Huang, Yanxia Wang, Lingyun Zeng, Jiezhi Yang, Xiuli Song, Wenwang Rao, Hehua Li, Yuping Ning, Hongbo He, Ting Li, Kai Wu, Fengjuan Chen, Fengchun Wu, Xiangyang Zhang

**Affiliations:** ^1^Department of Psychiatry, The Affiliated Brain Hospital of Guangzhou Medical University (Guangzhou Huiai Hospital), Guangzhou, China; ^2^Department of Scientific Research Center, Gansu Provincial Maternity and Child-Care Hospital, Lanzhou, China; ^3^Department of Psychiatry, Shenzhen Kangning Hospital, Shenzhen, China; ^4^Department of Psychiatry, Shenzhen Health Development Research Center, Shenzhen, China; ^5^Clinical Psychology, Yantai Affiliated Hospital of Binzhou Medical University, Yantai, China; ^6^Unit of Psychiatry, Faculty of Health Sciences, University of Macau, Macau, China; ^7^The First School of Clinical Medicine, Southern Medical University, Guangzhou, China; ^8^Department of Biomedical Engineering, School of Materials Science and Engineering, South China University of Technology (scUT), Guangzhou, China; ^9^Department of Medical, Guangzhou Eighth People’s Hospital, Guangzhou Medical University, Guangzhou, China; ^10^Department of Psychiatry, Guangdong Engineering Technology Research Center for Translational Medicine of Mental Disorders, Guangzhou, China; ^11^CAS Key Laboratory of Mental Health, Institute of Psychology, Chinese Academy of Sciences, Beijing, China

**Keywords:** prevalence, somatic symptoms, anxiety, insomnia, Coronavirus Disease 2019

## Abstract

**Background:**

Anxiety has been a common mental state during the epidemic of Coronavirus Disease 2019 (COVID-19) and is usually closely related to somatization. However, no study on somatization in anxiety and its relationship with insomnia has been conducted. Therefore, this study aimed to identify the prevalence of anxiety, somatization and insomnia and explore the relationships between different psychological states in the general population during the COVID-19 outbreak.

**Methods:**

A total of 1,172 respondents were recruited from 125 cities in mainland China by an online questionnaire survey. All subjects were evaluated with the 7-item Generalized Anxiety Disorder (GAD-7) scale, the somatization subscale of the Symptom Checklist 90-Revised (SCL-90-R), and the 7-item Insomnia Severity Index (ISI).

**Results:**

The percentages of anxiety, somatization, and insomnia were 33.02%, 7.59%, and 24.66%, respectively. The prevalence of somatization was 19.38% in participants with anxiety. Compared to the anxiety without somatization group, the anxiety with somatization group had a significantly higher percentage of patients with a history of physical disease and insomnia, as well as higher GAD-7 scores and SCL-90 somatization subscores (all p < 0.001). The SCL-90 somatization subscores were positively correlated with age, history of physical disease, GAD-7 scores, and ISI scores (all p < 0.001). Furthermore, multivariate logistic regression showed that GAD-7 score, ISI score, and age were risk factors for somatization in the anxious population.

**Conclusions:**

Somatic and psychological symptoms were common in the general population during the COVID-19 outbreak. Somatic symptoms, anxiety, and insomnia are closely related, and improving anxiety and sleep quality may help relieve somatic symptoms.

## Introduction

The COVID-19 epidemic is a major public health emergency because of the disease’s rapid spread, wide range of infections and difficulty to prevent and control (1). According to the data released by the World Health Organization on April 5, the COVID-19 epidemic has affected more than 200 countries and regions around the world, with more than 1.13 million confirmed cases. During the period of epidemic pressure, people’s psychology, physiology and behavior change accordingly (2, 3). If an individual’s response is inappropriate or excessive, it can impact physical and mental health (4, 5), causing anxiety, fear, insomnia, or somatic symptoms. A previous study revealed that psychological factors could play a crucial role in public health strategies to control epidemics and pandemics (6). Understanding and studying the psychological state of the public during the turbulent epidemic period is of practical significance for helping psychological professionals and the government to provide psychological support and behavioral guidance.

Anxiety is a common mental state that affects psychology and the body in the short and long term. A recent national survey in China showed that anxiety has the highest prevalence of all mental disorders at 4.98% (7). Approximately 10–30% of the public worried about the possibility becoming infected with virus during an influenza outbreak (8–10). With the suspension of business and school and, in some cases, the closure of cities, personal anxiety becomes more complicated by the ongoing COVID-19 epidemic (8). Wang C et al. (10) found that during the early stage of the epidemic, over 28% of Chinese respondents suffered from moderate or severe anxiety symptoms. A cross-sectional survey showed that approximately 25% of college students experienced anxiety during the outbreak (8). To date, many studies have reported factors associated with anxiety during the outbreak. An increasing amount of evidence has revealed that being female, being a medical health worker, being acquainted with someone who has been infected and having organic disease elevate the risk of anxiety, while age greater than 40 years and family income stability decrease the risk (3, 8, 11, 12).

Somatization and anxiety are usually closely related. In the general population, anxiety disorders often overlap with a variety of somatic symptoms (13); however, the correlation between physical symptoms and anxiety is inconsistent. Some studies have identified that somatic symptoms are linked with psychological or physiological abnormalities, which could indicate a pathological condition (14). Meanwhile, Raffagnato A et al. reported that patients expressed their mental state through somatization symptoms (15). For example, approximately, 15%–45% of patients with persistent pain suffer from various degrees of anxiety (16, 17). In contrast, previous studies showed that physical symptoms may occur independent of anxiety symptoms (18, 19), but the mechanism underlying this finding is not yet clear. Furthermore, several demographic and socioeconomic risk factors for somatic symptoms have been revealed, such as gender (female), age (older), marital status, chronic illnesses, and employment status (20). Insomnia and anxiety symptoms were also considered risk factors for somatic symptoms in a general population of Hong Kong (21). Few studies have examined somatic symptoms during the COVID-19 epidemic. For instance, a survey of 1,255 nonmedical health workers found that the prevalence rate of somatization was 0.4% (12).

At present, several studies have reported the prevalence of anxiety, depression, insomnia, and other psychological states in the general population during the epidemic (1, 6–8, 10–12). However, there is a lack of research on the relationship between different mental states during this particular period, and no study on the prevalence of somatic symptoms in a population with anxiety and its relationship with insomnia or other mental states have not been reported. Therefore, we investigated the public’s mental health during the COVID-19 epidemic and aimed to (1) explore the prevalence of anxiety, somatization, and insomnia in a Chinese population; (2) examine the correlation between physical symptoms and psychological symptoms; and (3) provide a theoretical basis for intervention measures provided by psychologists and the government.

## Methods

### Participants and Setting

Using a cross-sectional design, an anonymous online questionnaire survey was used to assess the public’s psychological status during the COVID-19 epidemic. We adopted a snowball sampling strategy to focus on recruiting ordinary people who lived on the Chinese mainland during the COVID-19 epidemic. The online survey was initially distributed among college students, who were encouraged to pass it on to others. All respondents completed the survey in Chinese by using Ranxing Technology “SurveyStar” to reduce face-to-face interaction. Data collection was carried out during the COVID-19 epidemic (from February 14 to March 29, 2020). Inclusion criteria included (1) Chinese individuals living on the mainland and (2) willingness to complete the survey. Any subjects with psychotic disorders diagnosed in a medical institution were excluded.

This study was approved by the Ethics Committee of the Institute of Psychology of the Chinese Academy of Sciences. All participants provided informed consent before answering questions, and they could terminate the investigation at any time.

### Self-Measurement and Procedures

In this study, the structured questionnaire included the following sections: (1) sociodemographic characteristics; (2) history of exposure to COVID-19; (3) history of physical disease; and (4) psychological health status.

#### Sociodemographic Characteristics

Sociodemographic data included sex, age, weight, height, marital status, education level, occupational status (student or not a student), economic loss, smoking status, and drinking status. Furthermore, we asked an additional question: Do you have relatives or friends who have been infected with COVID-19? In addition, body mass index (BMI) was calculated based on height and weight. Age was divided into four groups: 20 years old or below, 21–30 years old, 31–40 years old, and older than 40 years old.

#### Self-Measurement

Anxiety, physical symptoms, and insomnia in the general population were assessed by the Chinese version of the 7-item Generalized Anxiety Disorder (GAD-7) scale, the Chinese version of the somatization subscale of the Symptom Checklist 90-Revised (SCL-90-R), and the 7-item Insomnia Severity Index (ISI). These self-reported scales have good reliability and validity for measuring psychological status (11, 22, 23).

The GAD-7 was used to screen for generalized anxiety and assess the severity of symptoms. Scores range from 0 (not present) to 21 points (extremely severe), and a score of ≥ 5 indicates the presence of anxiety symptoms (24, 25).

The ISI scale was used to evaluate the presence and severity of insomnia. The total score of the ISI scale varies from 0 (not present) to 28 points (severe), and a cut-off value of 8 indicates the presence of insomnia (26).

Somatic symptoms were identified by the somatization subscale of the SCL-90-R, which consists of 12 items (Cronbach’s α = 0.83) scored on a five-point Likert scale: none (1), mild (2), moderate (3), fairly severe (4), and severe (5). The total score of the subscale ranges from 12 (not present) to 60 points (extreme); the higher the score, the stronger the participant’s physical discomfort is. According to results normed on a Chinese population, a total score higher than 24 points (single factor score ≥ 2) indicates the presence of somatic symptoms (12, 27).

### Statistical Analysis

Data analysis was conducted using SPSS (version 18.0) software. Normally, distributed data are presented as the mean ± standard deviation (SD), and count data are presented as the number of people (%). Demographic and clinical variables were compared between groups by analysis of variance (ANOVA) for continuous variables and chi-squared tests for categorical variables. Since the original scores of all scales are not normally distributed (Kolmogorov-Smirnov test, p < 0.05), the data are expressed as medians with interquartile ranges. The nonparametric Mann-Whitney U test was used to compare each symptom between groups. Relationships between SCL-90 somatization subscores and demographic and clinical variables were examined using Spearman correlation analysis. Multivariate logistic regression analysis (“enter” model) was then used to assess the relevant factors associated with somatization symptoms. Somatic symptoms (yes or no) in anxious participants were regarded as the dependent variable, while factors that showed statistical significance in chi-squared tests and U tests were regarded as the independent variables. A P value <0.05 (two-tailed) was considered statistically significant.

## Results

### Sociodemographic Characteristics

Altogether, 1,172 respondents (812 females and 360 males) were recruited from 125 cities in China. Their average age was 28.39 ± 10.49 years. Among them, 287 people (24.49%) were aged ≤ 20 years, 443 people (37.80%) were aged 21–30 years, 285 people (24.32%) were aged 31–40 years, and 157 people (13.39%) were above 40 years old. Education levels were as follows: high school degree or below (167, 14.25%), technical or mechanical degree (223, 19.03%), bachelor’s degree (675, 57.59%), and master’s degree or above (107, 9.13%). More than half of the participants (587, 50.09%) were students. Approximately half of the participants (585, 49.91%) experienced economic loss during the epidemic period. A total of 153 participants (13.05%) had a history of physical diseases. Only 8 (0.68%) participants had relatives and friends who suffered from COVID-19. The detailed sociodemographic information is presented in **Table 1**.

**Table 1:** Sociodemographic data and scale scores of the anxiety and non-anxiety groups in the general population.

	All subjects	Non-anxiety group	Anxiety group	*F/χ2/Z*	*p-*value
	n = 1172	n = 785	n = 387		
Sex (M/F), n	360/812	237/548	132/255	0.309	0.591
Age, years				0.590	0.899
≤20, n(%)	287(24.49)	188(23.95)	99(25.58)		
21–30, n(%)	443(37.80)	296(37.71)	147(37.98)		
31–40, n(%)	285(24.32)	193(24.58)	92(23.77)		
40, n(%)	157(13.39)	108(13.76)	49(12.67)		
BMI, kg/m^2^, M (SD)	22.19(10.38)	22.15(3.37)	22.34(4.41)	0.752	0.386
Married, n(%)	463(39.51)	317(40.38)	146(37.73)	0.765	0.409
Education level				7.397	0.060
High school degree or below	165(14.2)	114(14.7)	51(13.1)		
Technical or mechanical degree	223(19.0)	161(22.5)	62(16.0)		
Bachelor’s degree	675(57.5)	431(54.9)	244(63.0)		
Master’s degree or above	107(9.1)	77(9.8)	30(7.7)		
Occupation (student), n(%)	587(50.09)	382(48.66)	205(52.97)	1.925	0.172
Economic loss (yes), n(%)	585(49.91)	367(46.75)	218(56.33)	14.301	0.001**
Relatives and friends suffered from COVID-19, n(%)	8(0.68)	4(0.51)	4(1.03)	1.050	0.451
History of physical disease, n(%)	153(13.05)	80(10.19)	73(18.86)	17.174	<0.001**
Smoking, n(%)	83(7.08)	53(6.75)	30(7.75)	0.394	0.303
Drinking, n(%)	213(18.17)	130(16.56)	80(20.67)	4.162	0.044*
Somatization, n(%)	89(7.59)	12(1.53)	75(19.38)	114.377	<0.001**
Insomnia, n(%)	289(24.66)	110(14.01)	179(46.25)	145.028	<0.001**
GAD-7 scores	2(0.6)	1(0.2)	7(6,10)	−28.363	<0.001**
SCL-90 somatization subscores	14(12.17)	12(13.15)	17(14.22)	−16.637	<0.001**
ISI scores	3(0.7)	2(0.5)	7(4.13)	−14.557	<0.001**

*P＜0.05, **P＜0.01.

### Prevalence of Anxiety, Somatization, Insomnia and Risk Factors

The prevalence of anxiety, somatization, and insomnia in a Chinese population during the COVID-19 epidemic was 33.02% (387/1172), 7.59% (89/1172), and 24.66% (289/1172), respectively. The proportion of somatization among participants with anxiety was 19.38% (75/387).

As shown in **Table 1**, there was no significant difference in demographic characteristics between the subjects with anxiety (n = 387) and subjects without anxiety (n = 785; all p > 0.05), except for economic loss (p = 0.001), history of physical disease (p < 0.001), and drinking (p = 0.044). Furthermore, the anxiety group had higher GAD-7 scores, SCL-90 somatization subscores, and ISI scores than the non-anxiety group (all p < 0.05). Multivariate logistic regression analyses found that participants who experienced economic loss had a 1.3 times higher probability of anxiety symptoms than participants without economic loss (OR = 1.30, 95% CI: 1.05–1.57, Wald x^2^ = 5.74, p = 0.017), while no significant difference in history of physical disease and drinking was found (p >0.05). Higher SCL-90 somatization subscores (OR = 1.10, 95% CI: 1.07–1.14, Wald x^2^ = 37.15, p < 0.001) and ISI scores (OR = 1.17, 95% CI: 1.13–1.19, Wald x^2^ = 104.25, p < 0.001) were associated with a greater risk of anxiety.

### Demographic and Clinical Factors Associated With Somatization and Non-Somatization in Participants With Anxiety Symptoms

The demographic data of participants with anxiety in the non-somatization group (n = 312) and the somatization (n = 75) group are presented in **Table 2**. There was a significant difference in age between the two groups (χ2 = 8.608, p = 0.035). Among those with anxiety, a higher proportion of subjects over 40 years old showed somatization, and a lower proportion of those aged 20 years or younger showed somatization. Compared to the non-somatization subgroup of anxious participants, a significantly higher percentage of anxious participants with somatization group had a history of physical disease (χ2 = 10.490, p = 0.030) and insomnia (χ2 = 39.316, p < 0.001). Mann-Whitney U test analysis showed that in the anxiety group, participants with somatization had higher GAD-7 scores, SCL-90 somatization subscores and ISI scores than nonsomatization participants (all p < 0.001). However, there was no significant difference in sex, BMI, marital status, education level, occupation, economic loss, smoking, and drinking between the participants in the somatization and non-somatization subgroups (all p > 0.05).

**Table 2:** Sociodemographic data and scale scores of the somatization subgroup and non-somatization subgroup of the anxiety group.

	Somatization group	Non-somatization group	*F/χ2/Z*	*p-*value
	n = 312	n = 75		
Sex (M/F), n	98/214	25/50	0.103	0.783
Age, years			8.608	0.035*
≤20, n(%)	83(26.60)	10(13.33)		
21–30, n(%)	130(41.67)	34(45.33)		
31–40, n(%)	68(21.79)	17(22.67)		
40, n(%)	31(9.94)	14(18.67)		
BMI, kg/m^2^, M(SD)	22.09(3.36)	22.52(3.57)	0.982	0.322
Married, n(%)	111(35.58)	35(46.67)	3.165	0.085
Education level			4.928	0.177
High school degree or below	15(20.0)	36(11.5)		
Technical or mechanical degree	9(12.0)	53(16.9)		
Bachelor’s degree	47(62.6)	197(63.1)		
Master’s degree or above	4(5.3)	26(8.3)		
Occupation (student), n(%)	172(55.13)	33(44.0)	3.006	0.094
Economic loss (yes), n(%)	169(54.17)	49(65.33)	5.283	0.710
Relatives and friends suffered from COVID-19, n(%)	4(1.28)	0	0.972	1.000
History of physical disease, n(%)	49(15.71)	24(32.0)	10.490	0.03*
Smoking, n (%)	28(8.97)	2(2.67)	3.364	0.090
Drinking, n (%)	88(28.21)	15(20.0)	0.116	0.876
GAD-7 scores	7(3.11)	10(7.16)	−7.008	<0.001**
SCL-90 somatization subscores	16(14,18)	27(24.31)	−13.488	<0.001**
ISI scores	7(3.11)	13(8.16)	−6.485	<0.001**
Insomnia, n(%)	120(38.46)	52(69.33)	39.316	<0.001**

*P＜0.05, **P＜0.01.

As shown in **Table 3**, multivariable logistic regression analysis was used to explore the risk factors for anxiety with somatization symptoms. The findings showed that GAD-7 scores (OR = 1.158, 95% CI: 1.085-1.236, Wald x^2^ = 19.446, p < 0.001), ISI scores (OR = 1.087, 95% CI: 1.036-1.140, Wald x^2^ = 11.697, p = 0.001), and age (OR = 1.743, 95% CI: 1.049-2.894, Wald x^2^ = 4.606, p = 0.032) were associated with somatization symptoms in anxiety participants, while no difference in history of physical disease was found (p > 0.05).

**Table 3:** Multivariable logistic regression analysis of related factors in the anxiety group.

	B	S.E,	Wald	p-value	OR (95% confidence interval)
GAD-7 score	0.147	0.033	19.446	<0.001**	1.158(1.085–1.236)
ISI score	0.083	0.024	11.697	0.001**	1.087(1.036–1.140)
Age	0.555	0.259	4.606	0.032*	1.743(1.049–2.894)
History of physical disease	0.562	0.372	2.287	0.13	1.755(0.847–3.638)

*P＜0.05, **P＜0.01.

**Figure 1:**
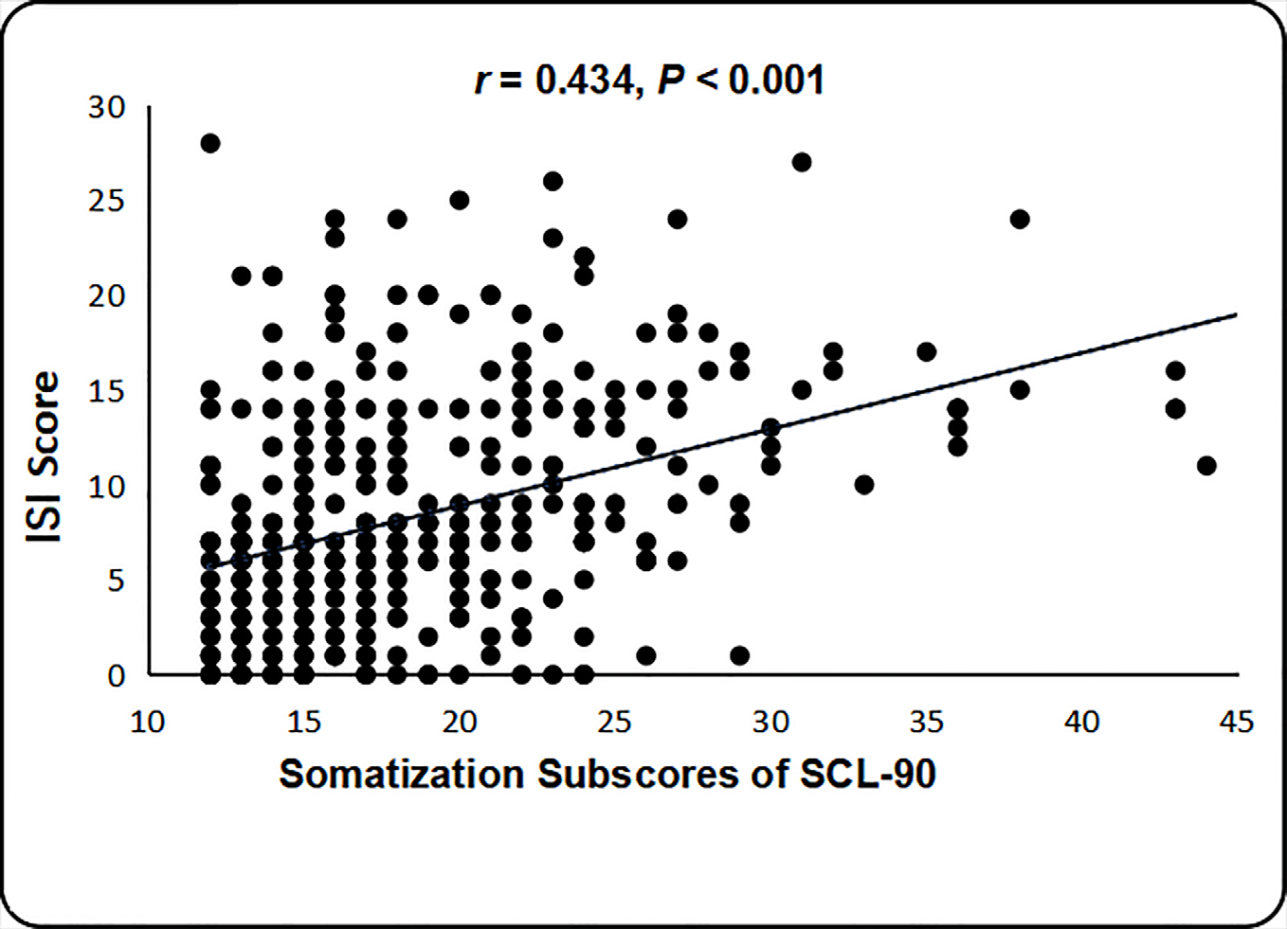
Relationship between SCL-90 somatization subscores and ISI scores in the anxiety group.

In the participants in the anxiety with somatization group, Spearman correlation analysis showed that SCL-90 somatization subscores were positively correlated with age (r = 0.192, p < 0.001), BMI (r =0.100, p = 0.049), history of physical disease (r = 0.236, p < 0.001), GAD-7 scores (r = 0.378, p < 0.001), and ISI scores (r = 0.434, p < 0.001) (**Figure 1**). However, in the anxiety without somatization group, SCL-90 somatization subscores were only positively correlated with GAD-7 scores (r = 0.197, p < 0.001) and ISI scores (r = 0.316, p < 0.001), and no significant correlation was found between SCL-90 somatization subscores and age, history of physical diseases, and BMI (all p > 0.05).

## Discussion

To the best of our knowledge, this is the first study to explore the prevalence of somatic symptoms and their related factors in a population with anxiety during the COVID-19 epidemic in mainland China. The main findings of this study are as follows: (1) the prevalences of anxiety, somatization, and insomnia were 33.02%, 7.59%, and 24.66%, respectively; (2) the prevalence of somatization in the population with anxiety was 19.38%; (3) patients in the anxiety with somatization group were more likely to have a history of physical disease and insomnia, older age, and higher GAD-7 scores; and (4) somatic symptoms were closely associated with anxiety and insomnia.

In our study, approximately one-third of the mainland Chinese respondents reported anxiety symptoms using the GAD-7 scale, which was consistent with most previous studies conducted during the COVID-19 outbreak. For example, a number of studies revealed that a relatively high proportion of the public (20–30%) experienced anxiety symptoms (8, 10). Due to the adverse effects of epidemics, such as fears of infection, limitations of social activities and daily life, and inevitable stress, mental health problems might arise (28). A survey including 600 subjects from China showed that only 6.33% of participants felt anxious (3). The differences in these studies might be partly attributed to differences in survey areas, interviewees, periods of the epidemic (initial, outbreak, and remission), measurements, etc. In addition, a consistent conclusion is that compared with the percentage (less than 5%) of the public with anxiety symptoms during the non-epidemic period (7, 13), a larger proportion of people present anxiety symptoms during the outbreak. The government and psychologists should constantly focus on the mental health of the public during this unique period. Furthermore, our study found that anxiety was associated with economic loss, a history of physical disease, and increased ISI scores, which is in line with previous studies (8, 29, 30). Interestingly, our study also demonstrated that anxious people were likely to have more somatic symptoms than people without anxiety symptoms.

Our survey further revealed a high proportion (19.38%) of somatic symptoms among the population with anxiety; this proportion was over 2 times more than the proportion of somatization for the whole sample (7.59%). In previous studies, the probability of somatization among people with anxiety fluctuated widely (ranging from 1.5% to 25%), depending on the different definitions of somatization used (31–34). Most studies defined physical symptoms in terms of both physical and psychological aspects (35). However, our study defined somatization as any discomfort, including unexplained and explained physical symptoms, that was equally strongly associated with anxiety (20). Using different self-rating scales instead of a standardized diagnosis to assess physical symptoms may be another reason for the inconsistent results (36, 37). Moreover, when people with physical diseases experience various physical discomforts, some of these symptoms can presented as psychological symptoms (38). Rosmalen and his panel reported that 11.8% of subjects in the general population with somatic symptoms suffered from depression or anxiety disorders (39). A 3-year follow-up study showed that the proportion of physical symptoms (both unexplained and explained symptoms) comorbid with any anxiety disorder was 17.4% (40). In addition, because findings regarding somatization show significant discrepancies and few studies have reported on somatization in anxiety, it is of great significance to investigate the factors related to the presence of somatic symptoms in people with anxiety.

Our study also found a significant difference in the history of physical diseases between the somatization and non-somatization subgroups of respondents with anxiety, which was similar to the findings of previous studies showing that chronic illness was highly correlated with somatic symptoms (12, 41–43). Unlike the findings from a population-based survey conducted in a Chinese general population, chronic illness was not related to somatic symptoms (21). The presence of different types of physical diseases may partly explain the differences between the two studies (21). Specifically, some diseases (osteoporosis, gout, arthritis, and low back pain) cause pain, while others (psychiatric illness, hypertension, diabetes, etc.) rarely cause pain.

Similar to an early study in Hong Kong (20), age was identified as a risk factor for somatization in individuals with anxiety. Nevertheless, other studies did not observe significant differences between different ages (21). Moreover, in contrast to previous studies (10, 44), no significant differences were observed in occupation status (students or others) and sex (male or female) between the two different anxiety subgroups, indicating that people with different occupations and genders suffered from similar physical and psychological states during this epidemic.

Compared with respondents without somatic symptoms, a greater proportion of respondents with anxiety and somatization suffered from insomnia and had higher ISI scores. Additionally, the correlation analysis further explained the positive correlation between insomnia scores and SCL-90 somatization subscores. A cross-sectional study involving 47,000 participants indicated that insomnia was closely related to somatic symptoms, similar to our results (45). We also demonstrated that the GAD-7 score was positively correlated with anxiety with comorbid somatization. The findings were consistent with those of several previous studies suggesting a close association between anxiety and somatization (12, 21, 44). A similar study (46) reported that compared with non-anxious people, anxiety patients were more sensitive to physical changes and had higher scores for psychological and physical symptoms, which could be explained by certain biological mechanisms (21). For example, an increasing level of anxiety can cause an increase in heart rate and blood pressure (47), which may also play a role in physical discomfort, such as the feeling of heart pressure. In addition, anxiety may trigger pain, which is related to increased muscle tension (48). Wilson and his colleagues also reported that anxiety may cause visceral allergies, resulting in exacerbated gastrointestinal discomfort (49). GAD-7 and ISI scores were regarded as predictive indicators of somatization in people with anxiety in the multivariate logistic regression analysis. Moreover, our research also demonstrated that somatization, anxiety, and insomnia coexist in the general population (13, 21). Anxiety may affect sleep quality by causing changes in hormone levels (such as increasing cortisol levels and decreasing melatonin synthesis) (50). Improving anxiety and sleep quality may help alleviate physical symptoms (21). However, due to the defects of cross-sectional studies, our study only reflected certain associations, and longitudinal studies are required to demonstrate causal relationships in the future.

Several limitations should be considered. First, due to the COVID-19 outbreak, a survey conducted by online questionnaires may have selection bias. These voluntary online surveys cannot artificially set the male-to-female ratio, and the imbalance between males and females may impact the results; thus, gender differences must be analyzed in the future. Moreover, the system cannot count the number of people who opened the connection but did not complete the questionnaire, so it is impossible to report the response rate. Second, clinical symptoms were assessed by a self-assessment scale instead of a standardized psychiatric diagnosis; however, the self-assessment scale has good reliability and validity. Third, this is a cross-sectional study, and it cannot explain internal causal relationships. Fourth, due to the requirements of epidemic prevention and control, COVID-19 patients (including asymptomatic infections) are admitted to hospital for isolation treatment, so we do not include diagnosed patients in this study, which may affect causal analyses. Fifth, in this study, we excluded any subjects who had been clearly diagnosed with psychotic disorders in a medical institution, which may have a certain impact on the incidence of anxiety, insomnia, and somatization.

In conclusion, our study demonstrated that anxiety, insomnia, and somatic symptoms were common in the general population during the COVID-19 epidemic. Moreover, somatic symptoms, anxiety, and insomnia are closely related, and improving anxiety and sleep quality may help relieve somatic symptoms. Therefore, we should pay attention to the mental state of the public during the COVID-19 epidemic and formulate relevant measures to intervene in cases of psychological problems.

## Data Availability Statement

All datasets presented in this study are included in the article/supplementary material.

## Ethics Statement

The studies involving human participants were reviewed and approved by the Ethics Committee of the Institute of Psychology of the Chinese Academy of Sciences. The patients/participants provided their written informed consent to participate in this study.

## Author Contributions

All authors contributed to the study design and data interpretation. FW and XZ were responsible for the management and oversight of the study. YH and YW were responsible for general omnibus data analyses and were the key contributing authors of the manuscript. LZ, JY, XS, HL, FC, and TL were responsible for all research interviews and clinical chart reviews associated with this study. YN and BH provided guidance on the design of the primary analyses. WR and KW assisted with all data collection, analysis, and writing of the manuscript. All authors contributed to the article and approved the submitted version.

## Funding

This research was supported by grants from the National Natural Science Foundation of China no. 31771074, the Science and Technology Plan Project of Guangdong no. 2019B030316001, the Science and Technology Project of Liwan District no. 201804011, and the Science and Technology Program of Guangzhou no. 201807010064, 201704020168, and 201804010259.

## Conflict of Interest

The authors declare that the research was conducted in the absence of any commercial or financial relationships that could be construed as a potential conflict of interest.
